# Characterisation of vaccine breakthrough infections of SARS-CoV-2 Delta and Alpha variants and within-host viral load dynamics in the community, France, June to July 2021

**DOI:** 10.2807/1560-7917.ES.2021.26.37.2100824

**Published:** 2021-09-16

**Authors:** François Blanquart, Clémence Abad, Joëvin Ambroise, Mathieu Bernard, Gina Cosentino, Jean-Marc Giannoli, Florence Débarre

**Affiliations:** 1 Infection Antimicrobials Modelling Evolution, UMR1137, INSERM, Université de Paris, Paris, France; 2 Centre for Interdisciplinary Research in Biology (CIRB), Collège de France, CNRS, INSERM, PSL Research University, Paris, France; 3 LBM BIOESTEREL–Biogroup–Plateau technique de Mouans-Sartoux, Mouans-Sartoux, France; 4 BPO-BIOEPINE–Biogroup–Plateau technique Chocolaterie, Levallois-Perret, France; 5 BIOLITTORAL – Biogroup – Plateau technique la Bastide, Sanary sur Mer, France; 6 UMR1173 INSERM, Université Paris-Saclay–UVSQ, Montigny-le-Bretonneux, France; 7 DYOMEDEA-NEOLAB-Biogroup – Plateau technique de la Sauvegarde, Lyon, France; 8 Institute of Ecology and Environmental Sciences of Paris (iEES-Paris, UMR 7618), CNRS, Sorbonne Université, UPEC, IRD, INRAE, 75252 Paris, France

**Keywords:** SARS-CoV-2, Delta, vaccination, breakthrough infection

## Abstract

We compared PCR results from SARS-CoV-2-positive patients tested in the community in France from 14 June to 30 July 2021. In asymptomatic individuals, Cq values were significantly higher in fully vaccinated than non-fully vaccinated individuals (effect size: 1.7; 95% CI: 1–2.3; p < 10^−6^). In symptomatic individuals and controlling for time since symptoms, the difference vanished (p = 0.26). Infections with the Delta variant had lower Cq values at symptom onset than with Alpha (effect size: −3.32; 95% CI: −4.38 to −2.25; p < 10^−6^).

The severe acute respiratory syndrome coronavirus 2 (SARS-CoV-2) variant of concern Delta (Phylogenetic Assignment of Named Global Outbreak (Pango) lineage designation B.1.617.2 and AY.* sublineages), first detected in India, spread across the world in 2021, and in particular in Europe in late spring to early summer 2021, where it displaced the previously dominant Alpha (B.1.1.7 and Q.* sublineages) variant. Delta was shown to spread faster than Alpha [1-3] and may be associated with higher virulence [4-6] and lower vaccine effectiveness [6,7] against symptomatic disease, especially with incomplete vaccination.

The Delta variant has spread in countries with high vaccination levels, and breakthrough infections have been reported, with quantification cycle (Cq) values (also called cycle threshold (Ct)) suggesting similar or lower viral loads in vaccinated compared to unvaccinated individuals, depending on the study [8-10]. A longitudinal study has confirmed similar Cq values in Delta-infected vaccinated and unvaccinated individuals in the first week after diagnosis or symptom onset, with later faster decline in vaccinated individuals [11]. Comparing Cq values in infections with the Delta variant and infections with previous variants requires controlling for time since infection when variants have different epidemiological dynamics. This is because viral load depends on time since infection, and the distribution of time since infection across individuals depends on whether the number of cases is growing or shrinking [12-16].

Here we use a large number of PCR tests done in the community in France at a time when the Delta variant was replacing other SARS-CoV-2 strains (mostly Alpha variant) to elucidate how vaccination status, infecting variant and the presence of symptoms impact viral loads.

## Description of the dataset

We studied the determinants of Cq values at the time of test and, for symptomatic individuals, as a function of the time since symptom onset, in 292,284 individuals tested from 14 June 2021 to 30 July 2021 by a large private laboratory group in the community in three regions of France (Bretagne, Île-de-France, Provence-Alpes-Côte d’Azur). These data included the result of the PCR test, the associated Cq value, the individual’s self-reported vaccine status (fully vaccinated for at least 2 weeks or not), whether the individual has been symptomatic and the time since onset of symptoms. Positive tests were screened for the L452R mutation, which characterises the Delta variant (9,343 positive tests had mutation information at this locus). If more than one test result was available for the same individual, we kept the last negative test if there were no positive tests, and the first positive test otherwise.

Consistent with the French vaccination campaign, vaccinated individuals in our dataset were on average (12 years older) older than non-vaccinated individuals (Supplement). The proportion of vaccinated individuals in the dataset (24%) was smaller than in the community (47.5% by 10 July 2021 [17]), reflecting the fact that the data are not surveillance-based. 

## Analysis of Cq values a function of vaccination status, variant and symptoms

**Figure 1:**
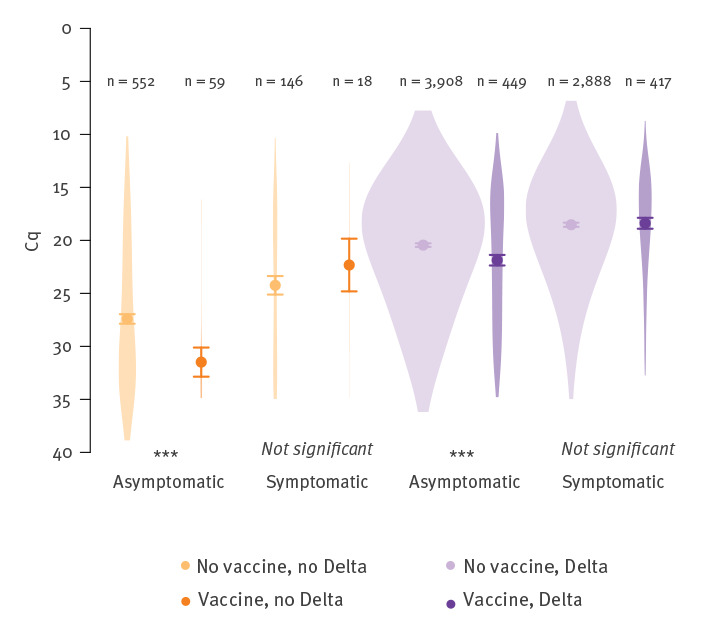
Distribution of Cq values, according to vaccine status, infecting variant and symptomatic status, France, 14 June–30 July 2021 (n = 8,437)

We first compared the Cq values (targeted at genes RdRp and N) of positive PCR tests, by vaccine status, presence of symptoms and infecting variant (Delta: presence of the L452R mutation), for the 8,437 individuals (3,174 female, 3,009 male, 2,254 unknown; 943 vaccinated, 7,494 unvaccinated; 6,284 < 40-years-old, 2,151 ≥ 40-years-old, two of unknown age) for whom all variables are available (Figure 1, Supplement). The Cq is the number of PCR cycles needed to detect a target; it is negatively correlated with viral load. 

Cq: quantification cycle.

*** The comparison of unvaccinated and vaccinated individuals is statistically significant with p < 0.001.

The widths of the violin plots reflect the number of tests; points and whiskers represent predicted values and 95% confidence intervals. Light colour: unvaccinated; dark colour: vaccinated; orange: non-Delta; purple: Delta; left side: asymptomatic at least until the test; right side: symptomatic.

The presence of symptoms was associated with significantly lower Cq values (linear model, effect size: −2.7; 95% confidence interval (CI): −3 to −2.5; adjusted p < 10^−6^, Tukey’s honest significance test). Infection with the Delta variant was also associated with lower Cq values compared with non-Delta (mostly Alpha) variants (effect size: −6.7; 95% CI: −7.1 to −6.3; adjusted p < 10^−6^). We note that time since infection is not controlled for here, but will in the analyses further down. Vaccinated individuals had significantly higher Cq values for both Delta and non-Delta asymptomatic infections than non-vaccinated individuals (effect size: 1.7; 95% CI: 1–2.3; adjusted p < 10^−6^), but this difference was not significant for symptomatic infections (p = 0.8). For non-Delta variants, this result may be a consequence of the sample size being too small (only n = 18 vaccinated symptomatic individuals infected with non-Delta variants).

## Analysis of Cq values controlling for the time since symptom onset

**Figure 2:**
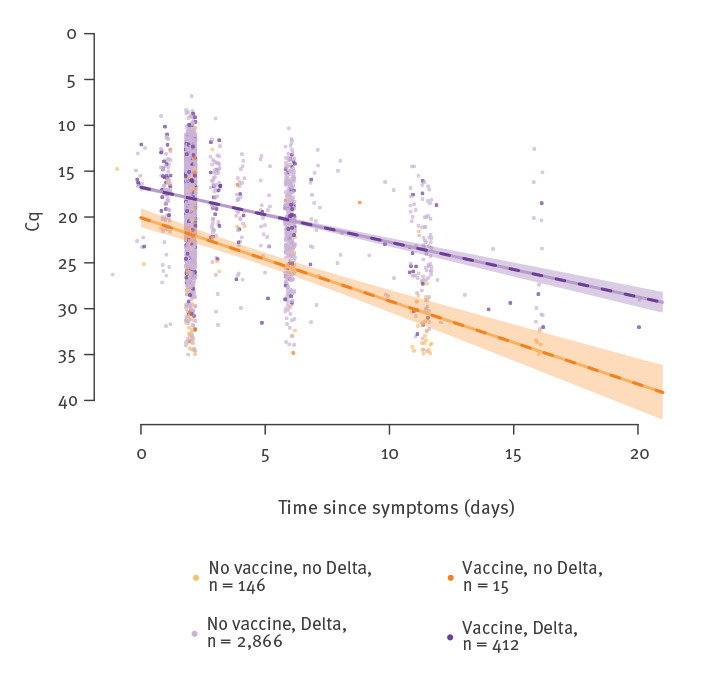
Regression of Cq values against time since symptom onset, for symptomatic individuals, by vaccine status and infecting variant, France, 14 June–30 July 2021 (n = 3,439)

The Cq values also depend on the time since infection of tested individuals. Controlling for time since infection avoids confounding factors that can arise when comparing Cq values of variants with different epidemiological dynamics [13,16], such as Delta (increasing numbers of infections) and Alpha (decreasing numbers of infections) in the early summer of 2021 in France. As noted above, vaccinated and unvaccinated individuals may also have different test-seeking behaviour, leading to different time since infection on the day of the test. We therefore added time since symptom onset as a continuous variable in the linear model for the subset of 3,439 symptomatic infections (Supplement). We first fitted a linear model predicting Cq value as a function of time since symptoms, vaccination status, variant (Delta or non-Delta) and all pairwise interactions (n = 3,439) (Figure 2). The Delta variant had a Cq difference of −3.32 on the day of symptom onset (95% CI: −4.38 to −2.25), compared to non-Delta (Alpha) (p < 10^−6^). The slope of Cq as a function of time was 0.6 (95% CI: 0.54–0.66) per day for Delta and 0.92 (95% CI: 0.73–1.1) for non-Delta variants (p < 10^−6^). Vaccine status did not significantly alter either the Cq at symptom onset (p = 0.256) or the slope of Cq as a function of time since symptom onset (p = 0.947).

Cq: quantification cycle.

Lines: predicted values; shaded areas: 95% confidence intervals. The vaccination effect was not significant and therefore removed from the linear model used in this figure.

### Ethical statement

Under French law, the use of research data that do not compromise patient privacy, as is the case with our dataset, is allowed by the French Commission Nationale Informatique et Libertés, and therefore our study did not require approval by an institutional review board.

## Discussion

Our dataset is unique for France because variant information and vaccine status data have not been linked yet in public datasets, and quantification values are not reported at the national level (only positive/negative test results are). Our results are in line with a retrospective cohort study which found lower Cq values with Delta and longer duration of infection with low Cq [5]. Regarding vaccine breakthroughs, our results confirm studies that found similar Cq values among fully vaccinated individuals and those who were not, with the majority of infections caused by the Delta variant [8,9]. Two recent studies examined the viral load in vaccinated compared with unvaccinated individuals [11,18]. Both found that viral loads declined faster in vaccinated individuals. One study found similar viral loads in vaccinated and unvaccinated individuals until 6 days post symptom onset (or diagnosis), then faster decline in vaccinated individuals [11]. The other, a systematic longitudinal study with small sample size, found that vaccinated individuals had a faster decline in viral load already from the day of the peak [18]. The different statistical models used in the two studies may explain the difference. We did not find in our data any difference in the Cq dynamics of symptomatic vaccinated and unvaccinated individuals, but we had little statistical power to detect differences in slope late in infection as in [11] because of a limited number of individuals presenting more than 6 days after symptom onset.

Limitations of our study stem from the way the data were collected. Reasons for seeking a SARS-CoV-2 PCR detection test are unknown, may vary among individuals and across time, and cannot be controlled for. Reasons for testing may vary between vaccinated and non-vaccinated individuals. This may especially be the case since France introduced a ‘sanitary passport’, requiring a proof of either full vaccination or a negative test for specific events, which may artificially inflate the proportion of negative tests among non-vaccinated individuals. Conversely, vaccinated individuals may get tested only if they have good reasons to suspect an infection. Reasons for seeking a test may also vary if symptoms differ depending on the infecting variant. Symptom and vaccine information are self-reported. Another limitation of our dataset is the lack of longitudinal data (each individual has only one Cq measure). Although our analysis takes advantage of a large sample size, additional data on randomly sampled individuals or systematic longitudinal surveys would complement our findings and shed more light on the impact of vaccination and variants on viral loads.

Finally, Cq values are a proxy: they are linked to viral load, and there is some evidence that viral load is associated with probability of transmission, although with considerable variation between individuals [19-21].

## Conclusion

Epidemic control may require similar measures for *symptomatic* PCR-positive vaccinated individuals as for non-vaccinated infected individuals. This remains true even if there is indeed a late faster viral load decline in vaccinated individuals, as most transmission would already have taken place.

## Supplementary Data

82000 Supplement

## References

[1] Campbell F Archer B Laurenson-Schafer H Jinnai Y Konings F Batra N Increased transmissibility and global spread of SARS-CoV-2 variants of concern as at June 2021. Euro Surveill. 2021;26(24):2100509. 10.2807/1560-7917.ES.2021.26.24.2100509 34142653 PMC8212592

[2] Mlcochova P Kemp S Dhar MS Papa G Meng B Ferreira IATM SARS-CoV-2 B.1.617.2 Delta variant replication and immune evasion. Nature. 2021. 10.1038/s41586-021-03944-y 34488225 PMC8566220

[3] Alizon S Haim-Boukobza S Foulongne V Verdurme L Trombert-Paolantoni S Lecorche E Rapid spread of the SARS-CoV-2 Delta variant in some French regions, June 2021. Euro Surveill. 2021;26(28):2100573. 10.2807/1560-7917.ES.2021.26.28.2100573 34269174 PMC8284044

[4] Fisman DN, Tuite AR. Progressive increase in virulence of novel SARS-CoV-2 variants in Ontario, Canada. medRxiv 2021.07.05.21260050. Preprint. http://dx.doi.org / 10.1101/2021.07.05.21260050

[5] Ong SWX Chiew CJ Ang LW Mak T-M Cui L Toh MPHS Clinical and virological features of SARS-CoV-2 variants of concern: a retrospective cohort study comparing B.1.1.7 (Alpha), B.1.315 (Beta), and B.1.617.2 (Delta). Clin Infect Dis. 2021;ciab721. 34423834 10.1093/cid/ciab721 PMC8522361

[6] Sheikh A McMenamin J Taylor B Robertson C Public Health Scotland and the EAVE II Collaborators. SARS-CoV-2 Delta VOC in Scotland: demographics, risk of hospital admission, and vaccine effectiveness. Lancet. 2021;397(10293):2461-2. 10.1016/S0140-6736(21)01358-1 34139198 PMC8201647

[7] Lopez Bernal J Andrews N Gower C Gallagher E Simmons R Thelwall S Effectiveness of Covid-19 Vaccines against the B.1.617.2 (Delta) Variant. N Engl J Med. 2021;385(7):585-94. 10.1056/NEJMoa2108891 34289274 PMC8314739

[8] Brown CM Vostok J Johnson H Burns M Gharpure R Sami S Outbreak of SARS-CoV-2 infections, including COVID-19 vaccine breakthrough infections, associated with large public gatherings - Barnstable County, Massachusetts, July 2021. MMWR Morb Mortal Wkly Rep. 2021;70(31):1059-62. 10.15585/mmwr.mm7031e2 34351882 PMC8367314

[9] Public Health England (PHE). SARS-CoV-2 variants of concern and variants under investigation in England. Technical briefing 20. London: PHE; 2021. Available from: https://assets.publishing.service.gov.uk/government/uploads/system/uploads/attachment_data/file/1009243/Technical_Briefing_20.pdf

[10] Elliott P, Haw D, Wang H, Eales O, Walters CE, Ainslie KEC, et al. REACT-1 round 13 final report: Exponential growth, high prevalence of SARS-CoV-2 and vaccine effectiveness associated with Delta variant in England during May to July 2021. London: Imperial College; 2021. Available from: https://spiral.imperial.ac.uk/bitstream/10044/1/90800/2/react1_r13_final_preprint_final.pdf 10.1126/science.abl9551 PMC10763627 34726481

[11] Chia PY, Ong S, Chiew CJ, Ang LW, Chavatte JG, Mak TM, et al. Virological and serological kinetics of SARS-CoV-2 Delta variant vaccine-breakthrough infections: A multi-center cohort study. medRxiv. 2021.07.28.21261295. Preprint. http://dx.doi.org / 10.1101/2021.07.28.21261295 PMC8608661 34826623

[12] Alizon S, Selinger C, Sofonea MT, Haim-Boukobza S, Giannoli J-M, Ninove L, et al. Epidemiological and clinical insights from SARS-CoV-2 RT-PCR cycle amplification values. medRxiv. 2021.03.15.21253653. Preprint. http://dx.doi.org / 10.1101/2021.03.15.21253653

[13] Hay JA Kennedy-Shaffer L Kanjilal S Lennon NJ Gabriel SB Lipsitch M Estimating epidemiologic dynamics from cross-sectional viral load distributions. Science. 2021;373(6552):eabh0635. 10.1126/science.abh0635 34083451 PMC8527857

[14] Cosentino G Bernard M Ambroise J Giannoli J-M Guedj J Débarre F SARS-CoV-2 viral dynamics in infections with Alpha and Beta variants of concern in the French community. J Infect. 2021;S0163-44532100374-1. 34329672 10.1016/j.jinf.2021.07.031

[15] Althaus CL, Baggio S, Reichmuth ML, Hodcroft EB, Riou J, Neher RA, et al. A tale of two variants: Spread of SARS-CoV-2 variants Alpha in Geneva, Switzerland, and Beta in South Africa. medRxiv. 2021.06.10.21258468. Preprint. http://dx.doi.org / 10.1101/2021.06.10.21258468

[16] Hay JA, Kennedy-Shaffer L, Mina MJ. Viral loads observed under competing strain dynamics. medRxiv. 2021.07.27.21261224. Preprint. http://dx.doi.org / 10.1101/2021.07.27.21261224

[17] Santé Publique France. Données relatives aux personnes vaccinées contre la Covid-19. [Data relating to people vaccinated against Covid-19]. Paris: French Government. [Accessed: 2021-08-09]. French. Available from: https://www.data.gouv.fr/en/datasets/donnees-relatives-aux-personnes-vaccinees-contre-la-covid-19-1/

[18] Kissler SM, Fauver JR, Mack C, Tai CG, Breban MI, Watkins AE, et al. Viral dynamics of SARS-CoV-2 variants in vaccinated and unvaccinated individuals. medRxiv. 2021.02.16.21251535. Preprint. http://dx.doi.org / 10.1101/2021.02.16.21251535 PMC8693673 34941024

[19] Lee LYW Rozmanowski S Pang M Charlett A Anderson C Hughes GJ SARS-CoV-2 infectivity by viral load, S gene variants and demographic factors and the utility of lateral flow devices to prevent transmission. Clin Infect Dis. 2021;ciab421. 33972994 10.1093/cid/ciab421 PMC8136027

[20] Marc A, Kerioui M, Blanquart F, Bertrand J, Mitjà O, Corbacho-Monné M, et al. Quantifying the relationship between SARS-CoV-2 viral load and infectiousness. medRxiv. 2021.05.07.21256341. Preprint. http://dx.doi.org / 10.1101/2021.05.07.21256341 PMC8476126 34569939

[21] Lyngse FP, Mølbak K, Træholt Franck K, Nielsen C, Skov RL, Voldstedlund M, et al. Association between SARS-CoV-2 transmissibility, viral load, and age in households. medRxiv. 2021.02.28.21252608. Preprint. http://dx.doi.org / 10.1101/2021.02.28.21252608

