# Authors’ correction for Euro Surveill. 2021;26(37)

**DOI:** 10.2807/1560-7917.ES.2021.26.43.211028c

**Published:** 2021-10-28

**Authors:** Eurosurveillance editorial team

**Affiliations:** 1 European Centre for Disease Prevention and Control (ECDC), Stockholm, Sweden

In the article ‘Characterisation of vaccine breakthrough infections of SARS-CoV-2 Delta and Alpha variants and within-host viral load dynamics in the community, France, June to July 2021’ by Blanquart et al. published on 16 September 2021, the affiliation ‘Infection Antimicrobials Modelling Evolution, UMR1137, INSERM, Université de Paris, Paris, France’ was removed for François Blanquart and the remaining affiliations were renumbered. These corrections were made on 27 October 2021 upon request from the authors.

