# Supplementary material for: Characterisation of vaccine breakthrough infections of SARS-CoV-2 Delta and Alpha variants and within-host viral load dynamics in the community, France, June to July 2021
Source: Euro Surveill. 2021 Sep 16;26(37):2100824. doi: 10.2807/1560-7917.ES.2021.26.37.2100824 (PMC8447828; doi:10.2807/1560-7917.ES.2021.26.37.2100824)
Supplement: Supplement [file 21-00824_DEBARRE_Supplement.pdf]

Supplement

François Blanquart, Clémence Abad, Joëvin Ambroise, Mathieu Bernard, Gina Cosentino, Jean-Marc Giannoli, Florence Débarre.

This supplementary material is hosted by *Eurosurveillance* as supporting information alongside the article "*Characterisation of vaccine breakthrough infections of SARS-CoV-2 Delta and Alpha variants and within-host viral load dynamics in the community, France, June to July 2021*", on behalf of the authors, who remain responsible for the accuracy and appropriateness of the content. The same standards for ethics, copyright, attributions and permissions as for the article apply. Supplements are not edited by *Eurosurveillance* and the journal is not responsible for the maintenance of any links or email addresses provided therein.

Age distribution of 8,437 individuals included in the first analysis (not controlled for time since symptom onset)

Age category	N vaccinated	N unvaccinated
09	1	263
19	76	1463
29	220	2644
39	171	1446
49	131	805
59	125	567
69	96	194
79	82	66
89	32	36
90+	8	9
Other/Unknown	1	1

Sex of 8,437 individuals included in the first analysis (not controlled for time since symptom onset)

Sex	N vaccinated	N unvaccinated
F	394	2780
M	330	2679
Unknown	219	2035

Age distribution of 3,439 individuals included in the second analysis (controlled for time since symptom onset)

Age category	N vaccinated	N unvaccinated
19	31	554
29	116	1118
39	72	609
49	51	330
59	69	211
69	37	74
79	33	19
89	16	11
90+	1	2
Other/Unknown	1	0

Sex of 3,439 individuals included in the second analysis (controlled for time since symptom onset)

Sex	N vaccinated	N unvaccinated
F	189	1378
M	142	1288
Unknown	96	346

Variables

name	type	description
`sympf`	factor (0, 1)	Whether the individual is fully vaccinated (1) or not (0)
`deltaf`	factor (0, 1)	Whether the L452R is detected (1) or not (0)
`vacf`	factor (0, 1)	Whether the individual is fully vaccinated (1) or not (0)
`Ct`	numeric	Ct value, RdRp gene
`tSS`	numeric	Time since symptom onset (days)

Linear Model not controlling for time since symptom onset

```
mdlAov$call

## aov(formula = Ct ~ vacf * sympf * deltaf, data = tmp)

summary(mdlAov)

##                     Df Sum Sq Mean Sq  F value   Pr(>F)
## vacf                 1    351     351   12.108 0.000505 ***
## sympf                1  15200   15200  524.896  < 2e-16 ***
## deltaf               1  32358   32358 1117.379  < 2e-16 ***
## vacf:sympf           1    775     775   26.766 2.35e-07 ***
## vacf:deltaf          1    144     144    4.970 0.025819 *
## sympf:deltaf         1    359     359   12.412 0.000429 ***
## vacf:sympf:deltaf    1    227     227    7.837 0.005130 **
## Residuals         8429 244096      29
## ---
## Signif. codes:  0 '***' 0.001 '**' 0.01 '*' 0.05 '.' 0.1 ' ' 1

thsd

##   Tukey multiple comparisons of means
##     95% family-wise confidence level
##
## Fit: aov(formula = Ct ~ vacf * sympf * deltaf, data = tmp)
##
## $vacf
##          diff       lwr      upr     p adj
## 1-0 0.6470032 0.2825148 1.011492 0.0005046
##
## $sympf
##          diff       lwr       upr p adj
## 1-0 -2.726566 -2.960009 -2.493124     0
##
## $deltaf
##         diff       lwr       upr p adj
## 1-0 -6.72394 -7.121566 -6.326314     0
##
## $`vacf:sympf`
##                diff        lwr        upr     p adj
```

```
## 1:0-0:0  1.6816459  1.0341760  2.3291159 0.0000000
## 0:1-0:0 -2.5080529 -2.8335307 -2.1825750 0.0000000
## 1:1-0:0 -2.7595748 -3.4541112 -2.0650384 0.0000000
## 0:1-1:0 -4.1896988 -4.8526381 -3.5267595 0.0000000
## 1:1-1:0 -4.4412207 -5.3445110 -3.5379303 0.0000000
## 1:1-0:1 -0.2515219 -0.9605014  0.4574576 0.7986775
##
## $`vacf:deltaf`
##                diff         lwr       upr     p adj
## 1:0-0:0  2.0758433   0.4153934  3.736293 0.0072293
## 0:1-0:0 -6.5769166  -7.1265213 -6.027312 0.0000000
## 1:1-0:0 -5.9746925  -6.6780558 -5.271329 0.0000000
## 0:1-1:0 -8.6527600 -10.2374675 -7.068052 0.0000000
## 1:1-1:0 -8.0505358  -9.6949058 -6.406166 0.0000000
## 1:1-0:1  0.6022242   0.1033012  1.101147 0.0104180
##
## $`sympf:deltaf`
##               diff       lwr       upr p adj
## 1:0-0:0 -3.824406 -5.040470 -2.608343     0
## 0:1-0:0 -7.225312 -7.822622 -6.628002     0
## 1:1-0:0 -9.324285 -9.933266 -8.715303     0
## 0:1-1:0 -3.400906 -4.500778 -2.301033     0
## 1:1-1:0 -5.499879 -6.606133 -4.393624     0
## 1:1-0:1 -2.098973 -2.417982 -1.779963     0
##
## $`vacf:sympf:deltaf`
##                    diff         lwr          upr     p adj
## 1:0:0-0:0:0   4.0748024   1.8402263   6.30937863 0.0000009
## 0:1:0-0:0:0  -3.1720965  -4.6903764  -1.65381653 0.0000000
## 1:1:0-0:0:0  -5.0857054  -8.9932302  -1.17818060 0.0020482
## 0:0:1-0:0:0  -6.9645855  -7.7063452  -6.22282583 0.0000000
## 1:0:1-0:0:0  -5.5406910  -6.5774897  -4.50389237 0.0000000
## 0:1:1-0:0:0  -8.8873726  -9.6453033  -8.12944188 0.0000000
## 1:1:1-0:0:0  -9.0238193 -10.0823280  -7.96531060 0.0000000
## 0:1:0-1:0:0  -7.2468989  -9.7636751  -4.73012268 0.0000000
## 1:1:0-1:0:0  -9.1605078 -13.5534270  -4.76758868 0.0000000
## 0:0:1-1:0:0 -11.0393880 -13.1792936  -8.89948235 0.0000000
## 1:0:1-1:0:0  -9.6154935 -11.8746836  -7.35630334 0.0000000
## 0:1:1-1:0:0 -12.9621750 -15.1077397 -10.81661041 0.0000000
## 1:1:1-1:0:0 -13.0986217 -15.3678571 -10.82938634 0.0000000
## 1:1:0-0:1:0  -1.9136089  -5.9890944   2.16187654 0.8466421
## 0:0:1-0:1:0  -3.7924891  -5.1676397  -2.41733844 0.0000000
## 1:0:1-0:1:0  -2.3685946  -3.9228736  -0.81431555 0.0001056
## 0:1:1-0:1:0  -5.7152761  -7.0992165  -4.33133582 0.0000000
## 1:1:1-0:1:0  -5.8517228  -7.4205671  -4.28287852 0.0000000
## 0:0:1-1:1:0  -1.8788802  -5.7330487   1.97528842 0.8194703
## 1:0:1-1:1:0  -0.4549857  -4.3766383   3.46666698 0.9999686
## 0:1:1-1:1:0  -3.8016672  -7.6589806   0.05564621 0.0567588
## 1:1:1-1:1:0  -3.9381139  -7.8655620  -0.01066581 0.0488069
## 1:0:1-0:0:1   1.4238945   0.6109881   2.23680092 0.0000031
```

```
## 0:1:1-0:0:1  -1.9227871  -2.3231923  -1.52238184 0.0000000
## 1:1:1-0:0:1  -2.0592338  -2.8996539  -1.21881362 0.0000000
## 0:1:1-1:0:1  -3.3466816  -4.1743701  -2.51899298 0.0000000
## 1:1:1-1:0:1  -3.4831282  -4.5926552  -2.37360130 0.0000000
## 1:1:1-0:1:1  -0.1364467  -0.9911733   0.71827990 0.9997296
```

Linear model controlling for time since symptom onset

Full model

```
mdl$call

## lm(formula = Ct ~ vacf * deltaf * tSS, data = dat.nodupl)

car::Anova(mdl)

## Anova Table (Type II tests)
##
## Response: Ct
##                  Sum Sq   Df  F value    Pr(>F)
## vacf                 27    1   1.2884  0.256427
## deltaf             3192    1 152.3081 < 2.2e-16 ***
## tSS                9487    1 452.6392 < 2.2e-16 ***
## vacf:deltaf           5    1   0.2289  0.632364
## vacf:tSS              0    1   0.0044  0.947141
## deltaf:tSS          211    1  10.0434  0.001542 **
## vacf:deltaf:tSS      44    1   2.1114  0.146299
## Residuals         71911 3431
## ---
## Signif. codes:  0 '***' 0.001 '**' 0.01 '*' 0.05 '.' 0.1 ' ' 1
```

Reduced model with significant effect only (used for the Figure)

```
mdl1$call

## lm(formula = Ct ~ deltaf + tSS + deltaf * tSS, data = dat.nodupl)

car::Anova(mdl1)

## Anova Table (Type II tests)
##
## Response: Ct
##             Sum Sq   Df F value    Pr(>F)
## deltaf        3231    1 154.156 < 2.2e-16 ***
## tSS           9512    1 453.905 < 2.2e-16 ***
## deltaf:tSS     228    1  10.872 0.0009863 ***
## Residuals    71987 3435
## ---
## Signif. codes:  0 '***' 0.001 '**' 0.01 '*' 0.05 '.' 0.1 ' ' 1
```
